# Estrogen replacement modulates voltage-gated potassium channels in rat presympathetic paraventricular nucleus neurons

**DOI:** 10.1186/1471-2202-14-134

**Published:** 2013-11-04

**Authors:** Seul Ki Lee, Pan Dong Ryu, So Yeong Lee

**Affiliations:** 1 Laboratory of Veterinary Pharmacology, College of Veterinary Medicine and Research Institute for Veterinary Science, Seoul National University, 1 Gwanak-ro, Gwanak-gu, 151-742, Seoul, Korea

**Keywords:** Estrogen, PVN-RVLM neuron, Kv4.2, Transient outward potassium current

## Abstract

**Background:**

The hypothalamic paraventricular nucleus (PVN) is an important site in the regulation of the autonomic nervous system. Specifically, PVN neurons projecting to the rostral ventrolateral medulla (PVN-RVLM) play a regulatory role in the determination of the sympathetic outflow in the cardiovascular system. In the PVN-RVLM neurons, the estrogen receptor β is expressed. However, to date, the effects of estrogen on PVN-RVLM neurons have not been reported. The present study investigated estrogen-mediated modulation of two voltage-gated potassium channel (Kv) subunits, Kv4.2 and Kv4.3, that are expressed predominantly in PVN neurons and the functional current of Kv4.2 and Kv4.3, the transient outward potassium current (I_A_).

**Results:**

Single-cell real-time RT-PCR analysis showed that 17β-estradiol (E2) replacement (once daily for 4 days) selectively down-regulated Kv4.2 mRNA levels in the PVN-RVLM neurons of ovariectomized female rats. There was no change in Kv4.3 levels. Whole-cell patch-clamp recordings demonstrated that E2 also diminished I_A_ densities. Interestingly, these effects were most apparent in the dorsal cap parvocellular subdivision of the PVN. E2 also shortened a delay in the excitation of the PVN-RVLM neurons.

**Conclusions:**

These findings demonstrate that E2 exerts an inhibitory effect on the functions of I_A_, potentially by selectively down-regulating Kv4.2 but not Kv4.3 in PVN-RVLM neurons distributed in a specific parvocellular subdivision.

## Background

The hypothalamic paraventricular nucleus (PVN) is an important site of neuroendocrine and autonomic control and has distinct subpopulations with different morphological and anatomical characteristics. The magnocellular cells project to the pituitary gland, and the parvocellular cells project to the median eminence, brain stem, and spinal cord [1]. Within the parvocellular cells, the presympathetic neurons project directly to the intermediolateral column in the spinal cord where the sympathetic preganglionic nuclei are concentrated or project indirectly via the rostral ventrolateral medulla (RVLM) to influence cardiovascular sympathetic outflows [2]. The RVLM contains vasomotor neurons, which regulate tonic and phasic vasomotor tone [3]. Several previous studies have revealed the important role of PVN-RVLM pathways in the regulation of the cardiovascular system [4-6].

Estrogen, a typical ovarian hormone, influences homeostatic function, reproduction, and the immune system [7,8]. Estrogen regulates gene transcription by binding the nuclear estrogen receptor, which contains two subtypes, α (ER-α) and β (ER-β) [9]. The two different subtypes have been shown to be expressed widely in rat brain and distributed differentially across different regions of the brain, of which PVN exclusively expresses ER-β [10]. The distributional pattern of ER-β in the PVN is similar to that of the parvocellular cells projecting to the brain stem or spinal cord [11,12]. This similarity was confirmed by the observation that PVN-RVLM neurons identified by retrograde tracing expressed ER-β [13]. The aforementioned studies potentially indicate that PVN-RVLM neurons could be major target cells for estrogen within the PVN [10-13]. However, the effects of estrogen on this specific cell population have not been studied.

Similar to other neurons, potassium channels control the firing activity of PVN-RVLM neurons [14,15]. Studies have demonstrated that the transient outward potassium current (I_A_) plays an important role in controlling the membrane excitability and firing discharge of PVN-RVLM neurons and that it contributes to remodeling neuronal excitation under pathological conditions [14,16]. I_A_ is encoded by homomultimeric or heteromultimeric complexes of voltage-gated potassium channel (Kv) subunits within identical subfamilies [17,18]. Among those, Kv1.4 and Kv4.3 have been suggested to be molecular correlates of I_A_ in PVN-RVLM neurons based on their strong immunoreactivities [14]. The recent observation that Kv4.3 mRNA expression decreased coincident to diminished I_A_ in hypertensive rats [19] supports the correlation of Kv4.3 and I_A_ in PVN-RVLM neurons. Our previous study showed that Kv4.2 and Kv4.3 mRNAs were abundant in PVN neurons and that the differential expression of Kv4.2 and Kv4.3 was parallel to the differences in the delay in the onset of the first action potential [20] that is known to be associated with I_A_ in PVN neurons [21]. This suggests that these subunits are closely related to I_A_ in PVN neurons. Taken together, Kv4.2 and Kv4.3 are most likely molecular components underlying I_A_ in the PVN, including PVN-RVLM neurons.

Several studies have reported estrogenic regulation of Kv4.2 or Kv4.3 transcript levels in tissues from distinct organs, such as the heart [22,23] and uterus [24], as well as in a hormone-producing brain cell line [25], indicating that Kv4.2 and Kv4.3 are widely expressed target molecules that are modulated by estrogen. Furthermore, these studies have shown that alternations in transcript levels of these subunits coincide with changes in I_A_.

Hence, the current study hypothesized that estrogen would modify Kv4.2 and Kv4.3 transcript levels in PVN-RVLM neurons and investigated changes in the mRNA levels of these subunits in the PVN-RVLM neurons of ovariectomized female rats (OVX-rat) by 17β-estradiol (E2) replacement using single cell real-time RT-PCR. Additionally, the study examined alternations in the current density and kinetic properties (activation and inactivation) of I_A_ using whole cell patch-clamp recording.

## Methods

### Animals

Sprague-Dawley female rats (*n* = 36, 7 wks old) were used (Orient Bio Inc., Kyonggi-do, Korea). The rats were maintained under a 12-hr light/dark cycle (lights on at 9:00 A.M.) and given free access to food and water until sacrifice. Anesthesia was induced by an intraperitoneal injection of Zoletil (25 mg/kg) and xylazine (10 mg/kg) for the surgical procedure. Animals were bilaterally ovariectomized (OVX). Every effort was made to minimize the number of animals and their suffering. The experimental protocols were performed in accordance with the guidelines of the Institutional Animal Care and Use Committee of Seoul National University and were approved by the Institute of Laboratory Animal Resources of Seoul National University (SNU-100917-1).

### Retrograde tracing

One week after OVX, the retrograde-transported fluorescent tracer, FluoSphere-Red (Molecular Probes, Eugene, OR, USA), was injected into the RVLM of OVX-rats to identify PVN-RVLM neurons, as previously described [26]. The injection point was, on average, 12.5 mm caudal, 1.7 mm lateral, and 8.0 mm ventral from bregma. To verify that dye was injected into the RVLM, serial medulla slices (100 μm thicknesses) of all animals subjected to retrograde tracing were examined after sacrifice.

### E2 treatment

Treatments began on the fourth day after the retrograde tracing process. Rats were divided into two groups: the oil-treated group (oil-group) and the 17β-estradiol-treated group (E2-group). The treatments were given once daily for four consecutive days prior to sacrifice and brain slice preparation; subcutaneous injections of corn coil (0.1 ml; Sigma-Aldrich, St. Louis, MO, USA) and 17β-estradiol-3-benzoate (25 μg/kg in 0.1 ml corn oil; Sigma-Aldrich) were administered to the oil-group and the E2-group, respectively. The quality of treatments was assessed by comparing serum E2 concentrations, body weight gain rates, and uterus weights between the two groups. Serum E2 concentrations were measured using the Estradiol ELISA kit (Labor Diagnostika Nord GmbH & Co. KG, Nordhorn, Germany; sensitivity, 10 pg/ml) according to the manufacture’s manual. Blood samples were collected from the abdominal vein of rats before sacrifice, allowed to cluster for 30–40 min at room temperature, and centrifuged for 25 min at 4°C. The serum layers were removed carefully into new tubes, and stored in -70°C until used for the ELISA assay. Body weight was measured before every treatment for all animals. The ratio of increased body weight on each day to the weight on the first day of treatment was used for the body weight gain rate. Uteri were isolated after sacrifice, weighed, and normalized to body weights on the day of sacrifice.

### Hypothalamic slice preparation

Hypothalamic brain slices were prepared within 2 hr after the fourth treatment as previously described [26] and used for single cell real-time RT-PCR or electrophysiological recording. After anesthesia, each rat was perfused transcardially with the ice-cold sucrose-rich artificial cerebrospinal fluid (ACSF) containing (in mM) 210 sucrose, 26 NaHCO_3_, 5 KCl, 1.2 NaH_2_PO_4_, 1.2 CaCl_2_, 2.4 MgCl_2_, and 10 glucose. The brains were briefly removed and dissected to two or three coronal slices (300 μm), including PVN regions, using a vibrating tissue slicer (Vibratome 1000 plus, Vibratome Company, St. Louis, MO, USA). The slices were incubated in an oxygenated normal ACSF containing (in mM) 126 NaCl, 26 NaHCO_3_, 5 KCl, 1.2 NaH_2_PO_4_, 2.4 CaCl_2_, 1.2 MgCl_2_, and 10 glucose for at least 1 hr at 30°C until used.

### Single-cell real-time RT-PCR

To gain RNAs of PVN-RVLM neurons for real-time RT-PCR, the hypothalamic brain slice was transferred to the same recording chamber as that used for electrophysiological recording, perfused continuously with an oxygenated ACSF (4–5 ml/min), and maintained at 30–32°C. The fluorescent-labeled PVN-RVLM neurons were visualized by combining differential interference contrast video microscopy with a fluorescence microscope installed with a green filter cube (WG, Olympus, Tokyo, Japan). The cells were approached with patch pipettes, using a micromanipulator (MP-225, Sutter instruments, Novato, CA, USA), that were pulled from borosilicate glass capillaries (1.7 mm diameter and 0.5 mm wall thickness) and filled with nuclease-free water (Qiagen, Valencia, CA, USA). After the whole cell configuration was achieved, a slight negative pressure was applied to pull the cytoplasm contents of each cell into the pipette, taking care to avoid including the nucleus. Each mixture of cytoplasm and water was immediately removed into a prepared microtube containing (μl) 4 of nuclease free water (Qiagen) and 1 of RNase Inhibitor (Applied Biosystems, Foster City, CA, USA). Reverse transcription for single-cell cDNA preparations was carried out using High Capacity cDNA Reverse Transcription Kits (Applied Biosystems) according to the manufacturer’s instructions.

PCR amplification was induced with a fraction of the single cell cDNA using the StepOnePlus System (Applied Biosystems) with SYBR Green detection for Kv4.2 and Kv4.3. SYBR Green detection was carried out as previously described with a minor modification [20]. The primer sequences were as follows: Kv4.2 (GenBank: NM031730) forward 5′-ACAAACGAAGGGCACAGAAGA-3′, reverse 5′-CATGTAGGCATTTGCACTTCCA-3′; Kv4.3 (GenBank: NM031739) forward 5′-AACGCAGGGCACAGAAGAAG-3′, reverse 5′-GGCATTGGAGCTCCCTGTT-3′; and β-actin (GenBank: NM031144) forward 5′-CATTGCTGACAGGATGCAGAA-3′ reverse 5′-AGAGCCACCAATCCACACAGA-3′. The real-time PCR reactions contained (in μl): 2 of the cDNA template, 0.2 of 10 μM of each forward and reverse primer, 0.4 of 50× Rox dye, 10 of 2× SYBR master mix (SYBR premix Ex Taq, Takara Bio Inc., Japan), and nuclease free water. The total volume was 20 μl. The thermal protocol was as follows: pre-denaturation at 95°C for 10 sec, amplification composed of 50 cycles of denaturation at 95°C for 5 sec and annealing at 60°C for 25 sec, and the melt curve step following the amplification, to verify the specificity of the PCR products. All samples were run in duplicate. Negative controls were assessed by running RT without the reverse transcript. Kv4.2 and Kv4.3 mRNA expression was compared between the oil-group and the E2-group using relative quantitative analysis [27]. The threshold cycle (Ct) value of Kv4.2 and Kv4.3 was normalized to that of a reference gene, β-actin, in identical cells. Final values were expressed as the n-fold of Kv4.2 or Kv4.3 transcript levels in the E2-group to those in the oil-group. Primer efficiencies for each target and reference gene were calculated from the formula E = 10^[-1/slope]^[28], where slope was obtained from the relationship between serial cDNA dilutions and Ct values, resulting in 98% for Kv4.2, 101% for Kv4.3, and 98% for β-actin. The Ct values of β-actin were similar across the cells (coefficient of variation = 3%), between the groups, and among plates.

### Electrophysiological recording

A brain slice was placed in a recording chamber and perfused, and the fluorescent-labeled PVN-RVLM neurons were visualized as described above (see Single-cell real-time RT-PCR). Patch pipettes were prepared from the same glass capillaries as above, but filled with the internal solution containing (in mM) 135 K-gluconate, 5 KCl, 20 HEPES, 0.5 CaCl_2_, 5 EGTA, and 5 MgATP (the pH was adjusted to 7.2 with KOH). The open resistance of the pipette ranged from 3 to 7 MΩ. The Axoclamp 2B amplifier (Molecular Devices, Foster City, CA, USA) was used for whole cell recordings. The signals were filtered at 1 kHz and digitized at 10 kHz using an analog-digital converter (Digidata 1320A, Molecular Devices) and pClamp software (Version 9.0, Molecular Devices). The liquid junction potential (~14.3 mV) was corrected for further analysis.

I_A_ recordings were obtained in voltage clamp mode. To isolate I_A_, the modified ACSF was used, containing (in mM) 109 NaCl, 26 NaHCO_3_, 5 KCl, 1.2 NaH_2_PO_4_, 3.6 MgCl_2_, and 10 glucose plus 0.2 CdCl_2_, 3 EGTA, 30 tetraethylamonium (TEA), and 0.5 μM tetrodotoxin (TTX) to block calcium channels, delayed rectifier potassium channels, and voltage-gated sodium channels. The protocols consisted of depolarizing step pulses, ranging from -70 mV to +25 mV for 400 ms in 5 mV increments, from conditioning pulse to -90 mV or to -40 mV for 340 ms, as described previously [14]. Currents obtained from two different conditioning pulses were subtracted digitally using pClamp software. To verify that the obtained K^+^ current was transient A-type, 4-aminopyridine (4-AP; 5 mM) was applied at the end of the recording. I_A_ amplitudes were used for the subsequent analysis of activation characteristics and current densities. Activation curves were plotted with normalized chord conductance, versus step potentials, obtained by dividing the current amplitude by differences between each step potential and reversal potential of K^+^ ions (obtained from the Nernst equation; -87.04 mV); they were then fitted with the Boltzmann equation using OriginPro 8 (OriginLab Corporation, Northampton, MA, USA). Current densities were calculated by dividing I_A_ amplitudes by cell capacitances. Inactivation characteristics of I_A_ were analyzed using the protocol of conditioning step pulses, ranging from -120 mV to -35 mV for 50 ms in 5 mV increments, and successive depolarizing pulses to -10 mV for I_A_ activation, as described previously [14]. Inactivation curves were plotted with normalized current amplitudes versus conditioning step potentials, and fitted with the Boltzmann equation. Differences in voltage dependences of I_A_ activation and inactivation between the oil-group and the E2-group were analyzed by comparing the half-activation potential (V_1/2_, at which half of the I_A_ is activated), the slope factor obtained from the activation curve, the half-inactivation potential (V_1/2_, at which half of the I_A_ is inactivated), and slope factor obtained from the inactivation curve.

To investigate the effects of E2 on the membrane excitability of PVN-RVLM neurons, delays to action potential generation were examined in current-clamp mode for both the oil- and E2-groups. The cells were hyperpolarized to near -90 mV, and depolarized by injecting a +45 pA current. The delay time was determined by measuring the duration from the point initiating depolarization to the peak of the first action potential. All drugs were obtained from Sigma-Aldrich except TTX (Tocris Bioscience, Bristol, UK).

### Statistical analysis

All values were expressed as means ± S.E.M. Comparisons between the oil-group and the E2-group were analyzed using an unpaired *t*-test. When necessary, analyses included two-way ANOVAs with fisher LSD post hoc test. Differences were considered significant at *P* < 0.05. All statistical analyses were conducted using OriginPro 8 (OriginLab) or SPSS Statistics 20 (IBM Corporation, Armonk, NY, USA).

## Results

### The effects of E2 treatments on OVX-rats

**Figure 1:**
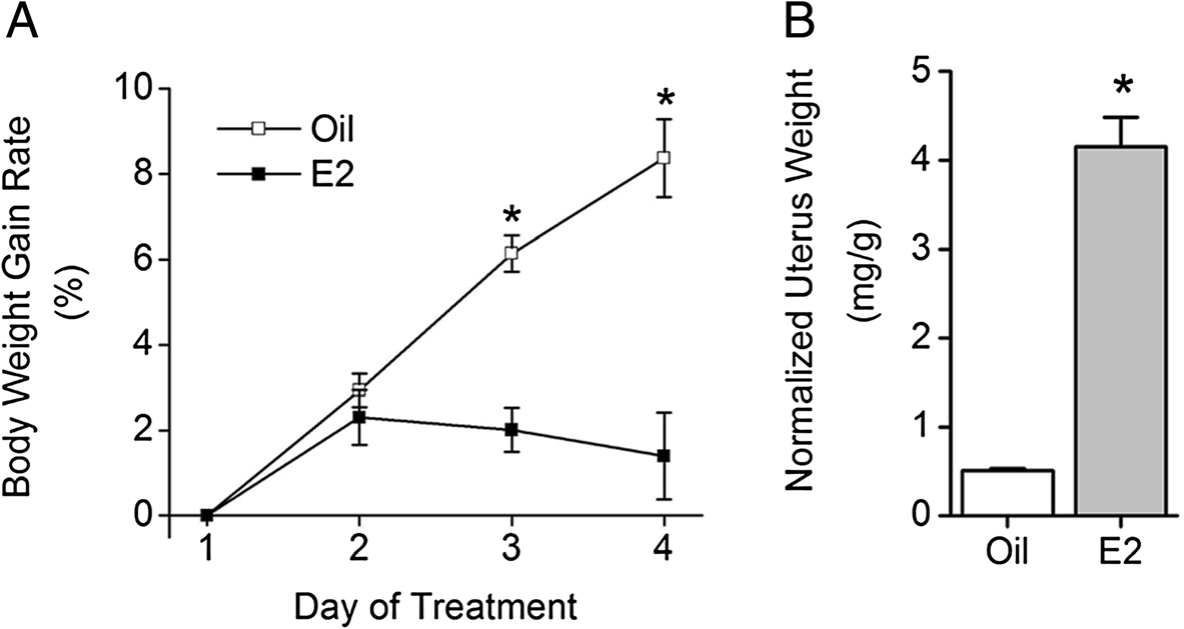
**Effects of E2 treatments on OVX rats.** OVX rats were treated with vehicle (corn oil, 0.1 ml) and 17β-estradiol-3-benzoate (25 μg/kg in 0.1 ml corn oil) for the oil-group and the E2-group, respectively. **A**, the body weight-gain rate was compared between the two groups by calculating the ratio of body weight increase each day to the weight on the first day of treatment. A significant difference appeared on the third and the fourth days (*P < 0.05). **B**, the mean uterus weight was compared between the two groups. Uteri were isolated after sacrifice and weighed. The weight was normalized to the body weight on the day of sacrifice. E2 treatment significantly increased uterus weight (*P < 0.05).

The mean serum E2 concentration measured after sacrifice was 25 ± 4 pg/ml in the oil-group (*n* = 9) and 59 ± 11 pg/ml in the E2-group (*n* = 9) and was significantly different between the two groups (*P* < 0.05). Suppression in body weight gain by the E2 treatment appeared significantly on the third day (6.14 ± 0.42% (*n* = 14) for the oil-group and 2.01 ± 0.52% (*n* = 10) for the E2-group, *P < 0.05); a larger effect was evident on the fourth day (8.37 ± 0.91% (*n* = 16) for the oil group and 1.39 ± 1.02 (*n* = 12) for the E2 group, *P < 0.05), as shown in Figure 1A. Uteri were isolated after sacrifice and normalized to body weights (uterus/body, mg/g). The mean normalized uterus weight was larger in the E2-group (4.15 ± 0.33 mg/g, *n* = 12, *P < 0.05) than in the oil-group (0.51 ± 0.02 mg/g, *n* = 16), as shown in Figure 1B.

### Retrograde labeling of PVN-RVLM neurons

**Figure 2:**
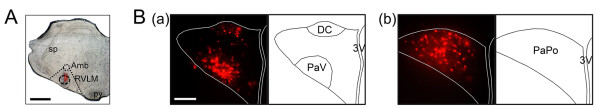
**Retrograde labeling of PVN-RVLM neurons. A**, RVLM injection site was verified with serial medulla slices including RVLM (100 μm thickness). The representative image shows that a fluorescent spot was confined to the triangular frame defined as RVLM [29]. Amb, nucleus ambiguus; sp, the spinal trigeminal nucleus; py, pyramidal tracts; scale bar = 1 mm. **B**, the fluorescent image illustrates that the labeled PVN-RVLM neurons were located in the medial region (a) and in the posterior region (b) of the PVN in the hypothalamic brain slices (300 μm thickness). The PVN-RVLM neurons are evident primarily in the dorsal cap (DC), the ventral parvocellular (PaV), and the posterior parvocellular (PaPo) subdivisions. 3 V, the third ventricle; scale bar = 200 μm.

To identify the PVN-RVLM neurons for recording and single-cell real-time RT-PCR, fluorescent tracer was injected into the RVLM of OVX-rats. After the animals were sacrificed, the serial medulla slices (100 μm thicknesses) were examined to determine that the dye was injected into the RVLM. A representative image of the medulla slice, including the RVLM, is illustrated in Figure 2A. A dye spot is present within the triangle frame, made by the nucleus ambiguus, the spinal trigeminal nucleus, and the pyramidal tracts, which was suggested as the guideline for RVLM location [29]. Figure 2B illustrates the fluorescent-labeled PVN-RVLM neurons in a rat hypothalamic brain slice. The PVN-RVLM neurons were located primarily in the three main PVN subdivisions: the dorsal cap (DC), the ventral parvocellular (PaV), and the posterior parvocellular (PaPo), which was consistent with the previous report [13].

### E2 effects on the mRNA expression density of Kv4.2 and Kv4.3 in single PVN-RVLM neurons

**Figure 3:**
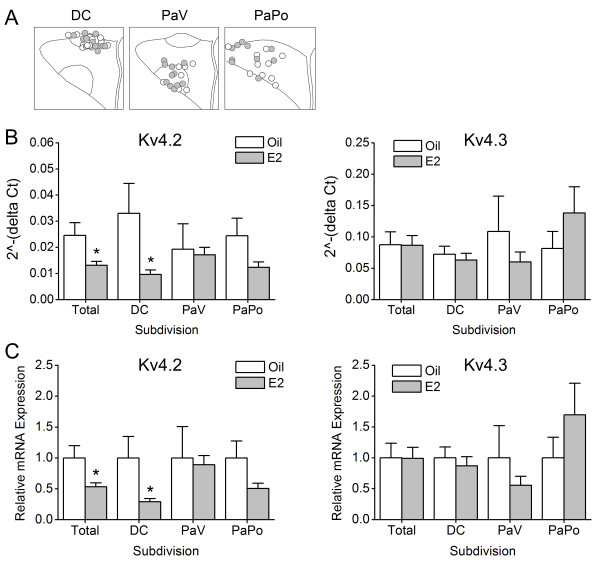
**E2 effects on mRNA expression density of Kv channel subunits in single PVN-RVLN neurons.** For relative quantification of Kv4.2 and Kv4.3 mRNA expression in the PVN-RVLM neurons between the oil- and the E2- groups, single-cell real-time RT-PCR was performed. **A**, the location of the PVN-RVLM neurons used for single-cell real-time RT-PCR in each subdivision. DC, the dorsal cap subdivision; PaV, the ventral parvocellular subdivision; PaPo, the posterior parvocellular subdivision. **B**, The mRNA expression density of the target genes of PVN-RVLM neurons was compared between the oil-group and the E2-group using the formula 2^-ΔCt^. **C**, the mRNA density of Kv4.2 and Kv4.3 was analyzed using the 2^-ΔΔCt^ method and was finally expressed as n-fold that of the oil-group. The Kv4.2 significantly decreased, 0.54 ± 0.06 fold, in the E2-group (*n* = 21, *P < 0.05) compared to that of the oil-group (*n* = 19). However, Kv4.3 was not expressed differently between the two groups. The mRNA expression densities of Kv4.2 and Kv4.3 were compared in the three different subdivisions: the DC, the PaV, and the PaPo. In the DC, Kv4.2 mRNA expression significantly decreased in the E2-group, compared to that of the oil group, and decreased more dominantly than in overall expression. In the PaV and PaPo, no significant differences were detected. Kv4.3 mRNA expressions were not statistically different between the two groups for any subdivisions.

To investigate the effect of E2 on the mRNA expression of Kv4.2 and Kv4.3 in rat PVN-RVLM neurons, single-cell real-time RT-PCR was performed. The mRNA expression density of the target genes of the PVN-RVLM neurons was compared between the oil-group and the E2-group using the formula 2^-ΔCt^ (Figure 3B), and then analyzed using the 2^-ΔΔCt^ method (Figure 3C), in which the mRNA expression density of Kv4.2 and Kv4.3 in the E2-group was expressed as n-fold that of the oil-group. The Kv4.2 decreased 0.54 ± 0.06 fold in the E2-group (*n* = 21; *P < 0.05) compared to that of the oil-group (*n* = 19; Figure 3C). However, Kv4.3 showed an unremarkable difference between the E2-group (1.00 ± 0.18 fold, *n* = 31) and the oil-group (*n* = 23). The mRNA expression density of Kv4.2 and Kv4.3 was analyzed according to the subdivisions where the PVN-RVLM neurons were distributed. The PVN-RVLM neurons used in each subdivision are illustrated in Figure 3A. As shown in Figure 3C, the cells in the DC displayed more prominent reductions in Kv4.2 mRNA expression by E2 (0.29 ± 0.05 fold, *n* = 8 in the E2-group and *n* = 4 in the oil group; *P < 0.05) than those in other subdivisions (in the PaV, 0.89 ± 0.15 fold, *n* = 8 in the E2-group and *n* = 6 in the oil group; in the PaPo, 0.51 ± 0.09 fold, *n* = 5 in the E2-group and *n* = 9 in the oil-group). In contrast, Kv4.3 mRNA expression densities exhibited the smallest differences in the DC (0.87 ± 0.15 fold, *n* = 12 in the E2-group; *n* = 9 in the oil group). In the PaV, Kv4.3 decreased 0.56 ± 0.15 fold in the E2-group (*n* = 9) compared to that of the oil group (*n* = 8), while in the PaPo, it increased 1.70 ± 0.51 fold in the E2-group (*n* = 10) compared to that of the oil group (*n* = 6), but the differences were not significant.

### E2 effects on I_A_ in PVN-RVLM neurons

To examine a change in electrophysiological properties of PVN-RVLM neurons as a result of E2, a whole cell clamp recording was performed with 67 PVN-RVLM neurons, 36 cells from the oil-group and 31 cells from the E2-group. The resting membrane potential (RMP), the input resistance (R_in_), and the membrane capacitance (C_m_) showed no differences between the two groups (RMP, −60.55 ± 0.67 mV in the oil-group and −60.36 ± 0.92 mV in the E2-group; R_in_, 492.89 ± 47.10 MΩ in the oil-group and 500.08 ± 42.73 MΩ in the E2-group; C_m_, 35.23 ± 2.16 pF in the oil-group and 36.19 ± 1.92 pF in the E2-group).

**Figure 4:**
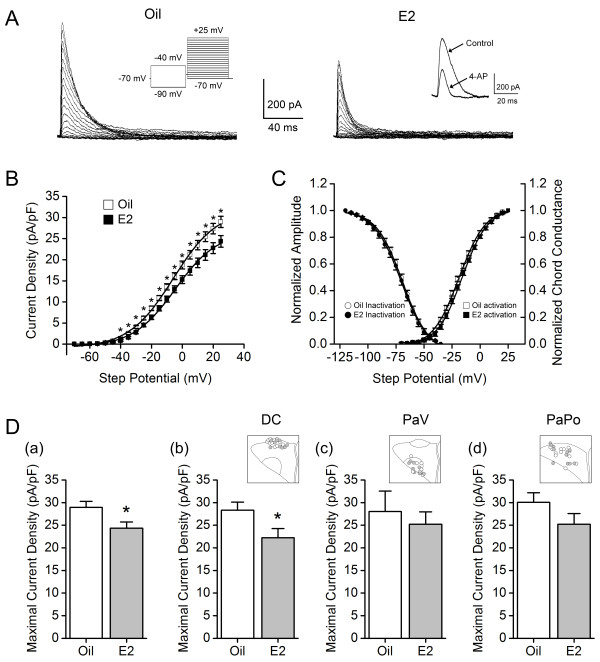
**E2 effect on I**_**A **_**in PVN-RVLM neurons. A**, representative images illustrate I_A_ isolated in PVN-RVLM neurons from the oil-group (left) and the E2-group (right). The left *inset* indicates the voltage protocol used to isolate I_A_. The right *inset* indicates the reduction in I_A_ by 4-aminopyridine (4-AP), the blocker of I_A_. **B**, Plots of I_A_ current densities versus command step potentials. The current density was calculated by dividing the current amplitude of I_A_ by cell capacitance. The E2 group (filled squares) displayed significant reductions in current densities of I_A_, compared to the oil-group (open squares). *P < 0.05. **C**, activation curve plotted with the mean normalized chord conductance (open squares for the oil-group and filled squares for the E2-group) and inactivation curve plotted with the mean normalized amplitude (open circles for the oil-group and filled circles for the E2-group). The curves were fitted with the Boltzmann equation. There were no significant differences in either the activation or the inactivation properties of I_A_. **D**, the maximal current density (at +25 mV step potential) was compared between the two groups. **(a)** In the E2-group, the maximal current density of I_A_ significantly decreased, compared to that of the oil-group. When the differences between the two groups were compared according to subdivisions, a significant difference was detected only in the DC **(b)**, not in either the PaV **(c)** or the PaPo **(d)**. *P < 0.05. *Insets* in **(b)**, **(c)** and **(d)** indicate the locations of the PVN-RVLM neurons used for I_A_ recording in each subdivision. DC, the dorsal cap subdivision; PaV, the ventral parvocellular subdivision; PaPo, the posterior parvocellular subdivision.

The study investigated possible I_A_ modulation by E2. To verify that the current recorded in this experiment was I_A_, 4-AP, the blocker of I_A_, was applied to the bath solution at the end of recording. When 30 cells were tested, 68% of I_A_ peak amplitudes were blocked by 5 mM 4-AP (Figure 4A, *inset* on right). The I_A_ current densities (the normalized current amplitude to cell capacitance) significantly decreased in the E2-group compared to those of the oil-group (Figure 4B, *P < 0.05). However, the voltage-dependence of I_A_ activation and inactivation was not different between the two groups (Figure 4C: for activation, V_1/2_ -18.84 ± 0.92 mV, slope factor 11.79 ± 0.38 (*n* = 36) in the oil-group and V_1/2_ -16.69 ± 1.04 mV, slope factor 11.22 ± 0.45 (*n* = 31) in the E2-group; for inactivation, V_1/2_ -69.14 ± 1.19 mV, slope factor 10.92 ± 0.41 (*n* = 33) in the oil-group and V_1/2_ -71.24 ± 1.71 mV, slope factor 10.84 ± 0.47 (*n* = 28) in the E2-group).

When the maximal current densities of I_A_ (at +25 mV step potential) were compared between the oil-group and the E2-group, the E2-group expressed significantly smaller current densities than those of the oil-group (24.3 ± 1.39 pA/pF for the E2 group and 29.0 ± 1.33 pA/pF for the oil-group, Figure 4D (a), *P < 0.05). The differential modulation in I_A_ current density by E2 was examined according to the different subdivisions, as shown in the mRNA expression analysis. A significant reduction in I_A_ current density occurred only in the DC (22.23 ± 2.04 pA/pF, *n* = 9 for the E2-group; 28.33 ± 1.79 pA/pF, *n* = 16 for the oil-group; Figure 4D (b), *P < 0.05); other subdivisions did not have similar significant changes (in the PaV, 25.20 ± 2.75 pA/pF (*n* = 9) in the E2-group and 28.03 ± 4.52 pA/pF (*n* = 6) in the oil-group, Figure 4D (c); in the PaPo, 25.22 ± 2.38 pA/pF, (*n* = 13) in the E2- group and 30.07 ± 2.11 pA/pF (*n* = 14) in the oil-group, Figure 4D (d)).

### E2 effects on delay of excitation in PVN-RVLM neurons

**Figure 5:**
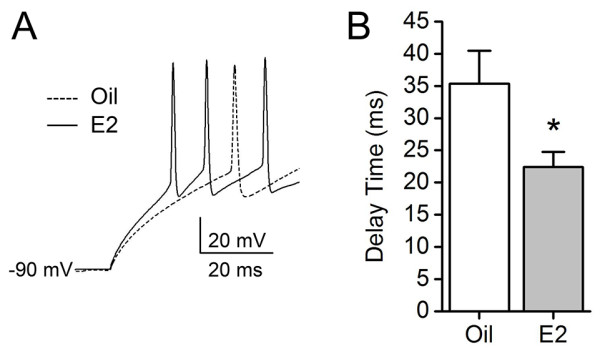
**E2 effects on delay of excitation in PVN-RVLM neurons. A**, a representative image shows a delay in the onset of the first action potential expressed in PVN-RVLM neurons from the oil-group (dashed line) and the E2-group (solid line). The cells were hyperpolarized to near -90 mV to remove I_A_ inactivation and depolarized by injecting a +45 pA current. **B**, The bar graph summarizes the differences in delay in the onset of the first action potential between the oil-group and the E2-group. The mean delay time of the E2-group significantly decreased, compared to that of the oil-group (*P < 0.05).

The study investigated E2 modulation of the membrane excitability of PVN-RVLM neurons by comparing delays in the onset of the first action potential between the oil-group and the E2-group. When hyperpolarized to near -90 mV and depolarized by injecting a +45 pA current, most cells generated action potentials. However, delay durations to the onset of the first action potential were different between the oil-group and the E2-group (Figure 5A). Delays significantly shortened in the E2-group (22.41 ± 2.37 ms, *n* = 17; *P < 0.05) compared to that of the oil-group (35.35 ± 5.10 ms, *n* = 23, Figure 5B). As the extent of hyperpolarization for removing I_A_ inactivation may influence delay durations, hyperpolarizations were compared between the two groups. There were no differences (-89.64 ± 0.64 mV in the oil group and 88.50 ± 1.05 mV in the E2-group).

## Discussion

The biophysical and pharmacological properties of the PVN-RVLM neurons, known as presympathetic neurons, have been extensively studied in physiological and pathological conditions [14,19,30]. It has also been demonstrated that ER, especially ER-β, is substantially expressed in the PVN-RVLM neurons [13] suggesting specific roles of E2 in the PVN-RVLM neurons. However, the effects of E2 on the cellular properties of PVN-RVLM neurons remain unknown. The present study investigated differences in the cellular properties of PVN-RVLM neurons between estrogen-deficient rats (the oil-group) and estrogen-replaced rats (the E2-group) following E2 treatment in vivo after ovariectomy. The investigation focused on Kv channel subunit expression and its functional current, I_A_, because I_A_ is one of the major currents expressed in PVN-RVLM neurons, and Kv channel subunits are modulated by altered physiological conditions [14,19]. The results showed that E2 significantly down-regulated the mRNA expression of Kv4.2, but not that of Kv4.3, and diminished I_A_ current density. Interestingly, this effect was only observed in the DC among three subdivisions (DC, PaV, and PaPo). E2 also shortened the delay in the onset of the first action potential that is known to be associated with I_A_ in PVN neurons [21]. These results suggest that E2 exerts an inhibitory effect on the function of I_A_, potentially by selectively down-regulating the transcript levels of Kv4.2 but not Kv4.3 in PVN-RVLM neurons distributed in the specific subdivision. However, some limitations should be considered in the present study. First, the electrophysiological recording and single cell real-time RT-PCR were performed in different cells rather than in the same cells due to the poor quality of the RNA samples following a long recording time and continuous exposure of the cells to multiple ion channel blockers. Therefore, a direct correlation between I_A_ and Kv4.2 in E2-mediated regulation cannot be confirmed. However, the observation that the down-regulation by E2 of the mRNA expression of Kv4.2 and the density of I_A_ appeared consistent indicates that Kv4.2 and I_A_ are closely related/associated in the cellular modulation of PVN-RVLM neurons. Second, the mechanism underlying E2-mediated regulation of Kv4.2 and I_A_ remains unclear. Future studies are warranted to confirm whether ER-β located in the PVN-RVLM neurons mediates the transcriptional regulation of Kv4.2. Third, the possibility that non-genomic actions of E2 contributed to Kv4.2 and I_A_ modulation in the PVN-RVLM neurons cannot be excluded because it has been shown that acute application of E2 in bath solution inhibits outward potassium currents, including I_A_[31]. E2 induces non-genomic events via membrane estrogen receptor or other E2-binding proteins, which activate second messenger cascades, including adenylate cyclase, phospholipase C, extracellular-signal-regulated kinase, and mitogen-activated protein kinase [32,33]. It was reported that inhibition of the I_A_ density by E2 replacement is reversed by application of a kinase inhibitor in gonadotropin-releasing hormone neurons of OVX-mice [34].

In spite of these limitations, this study is noteworthy because it is the first to reveal estrogenic modulation of cellular properties of PVN-RVLM neurons, particularly its target gene selectivity, for example, for Kv4.2 but not Kv4.3, and its regional selectivity in the DC subdivision. The selectivity for Kv4.2 is interesting because the major potential subunit for I_A_, to date, in the PVN-RVLM neurons has been considered to be Kv4.3 [14,19] because the latter is predominantly expressed in PVN-RVLM neurons [14]. However, our previous study found that Kv4.2 may also contribute to I_A_ in PVN neurons, despite its lower detection frequency compared to Kv4.3 [20]. The selective modulation of Kv4.2 by E2 suggests that even less dominantly expressed subunits could be involved in the cellular modulation or the functions of PVN-RVLM neurons and that gene modulation is not necessarily parallel to preponderance in the gene expression level. The target selectivity of E2 in the modulation of Kv4.2 and Kv4.3 is evident in distinct cell types. In heart cells, Kv4.3 was regulated by E2, whereas Kv4.2 was not [22,23]. In uterus cells, Kv4.2 and Kv4.3 were both regulated simultaneously by E2 during pregnancy [24,35]. In a GT1-7 cell line producing gonadotropin-releasing hormones, Kv4.2 was regulated by E2 [25]. Kv4.1 was regulated by E2 in the hypothalamic arcuate nucleus [36]. Therefore, estrogenic regulation has specific selectivity in molecular targeting, depending on distinct tissues or cells. This selectivity may be related to the differential expression of ER subtypes in distinct tissue. In the uterus and the heart, the expression of ER-α is greater than that of ER-β, which is uncommon in PVN-RVLM neurons.

Regional selectivity in E2-induced modulation of Kv4.2 mRNA expression suggests that the effect of E2 on the PVN-RVLM neurons is likely to be different on each subdivision. E2 significantly down-regulated Kv4.2 only in the DC. This finding is consistent with the recording data in which the I_A_ density was significantly down-regulated in the same division by E2, indicating that E2 modulation of PVN-RVLM neurons almost reflects the effects of E2 in the DC. From this result, we speculate that heterogeneous cells that have different sensitivities and responses to E2 exist in PVN-RVLM neurons and that these cells are clustered in a specific region. In this regard, the DC subdivision is likely to be the specific region where E2 exerts inhibitory effects on I_A_ functions of PVN-RVLM neurons, potentially by down-regulating Kv4.2. The specific modulation of E2 in the DC subdivision may be due to distinct properties of PVN-RVLM neurons distributed in this subdivision. For example, biochemical differences among subdivisions could account for this specific modulation. It has been reported that spinally-projecting PVN neurons which are distributed in similar regions to the PVN-RVLM neurons have different neurochemical characteristics according to subdivisions [37]. Dynorphin and oxytocin were detected in all subdivisions, but the expression of enkephalin and vasopressin was considerably lower in the DC compared to other subdivisions. Immunohistochemical studies showed that ER-β was abundantly double-labeled with oxytocin, whereas it was not with vasopressin or corticotropin-releasing hormone in PVN neurons [11,38]. Although such neurochemical differences have not been reported in PVN-RVLM neurons, the differences may be closely related to the regional-specific modulation by E2 in these neurons.

This study investigated the effects of E2 on the membrane excitability of PVN-RVLM neurons by observing changes in the delay of the onset of the first action potential between the oil-group and the E2-group, because I_A_ is known to exert a hyperpolarizing effect on membrane discharge to delay membrane excitation [39]. In agreement with E2-mediated down-regulation of I_A_, the results showed that the delay was shortened in the E2-group. The delay has been referred to as a representative characteristic of magnocellular PVN neurons because these neurons apparently express a stronger I_A_, coincidentally associated with a longer delay, than parvocellular PVN neurons [21]. Although the parvocellular PVN-RVLM neurons displayed much smaller delays than the magnocellular neurons, the delays are regulated to a significant extent by E2, and these are probably associated with the regulation of I_A_. This suggests that reductions in I_A_ and in the delay of the onset of the action potential by E2 influence the functions of PVN-RVLM neurons. However, the neuronal properties of PVN-RVLM neurons are determined not only by I_A_, but also by a variety of other factors. Regarding the intrinsic membrane property, other ionic currents besides I_A_, including voltage-gated calcium channels and small conductance calcium-activated potassium channels, participate in determining the excitability of PVN-RVLM neurons [15,40]. Studies have reported that E2 modulates these channels in other neuronal cells within the brain [41-43]. Research has also shown that neuronal activity is governed not only by intrinsic membrane properties, but also by the synaptic inputs of excitatory and inhibitory signals [30]. In addition, studies have demonstrated that receptors underlying the excitatory and inhibitory synaptic signals are also influenced by E2 in other brain regions [44,45].

The aforementioned raises the question of how E2-induced cellular modulation of PVN-RVLM neurons influences cardiovascular regulation. Studies have demonstrated that estrogen plays protective roles in the pathological development of, and the incidence of, cardiovascular disorders [46-48]. Accumulating evidence shows that the neuronal activity of PVN-RVLM neurons is elevated by enhancing the intrinsic membrane activity or by eliminating inhibitory synaptic inputs in cardiovascular diseases, such as hypertension and chronic myocardial infarction [16,26,49]. Therefore, the E2-mediated modulation of PVN-RVLM neurons shown in this study (i.e., the diminution in I_A_ density and the likely enhancement of membrane excitability) seems to be controversial with respect to the protective effects of E2 on the cardiovascular system because the modified pattern is more likely to reinforce the increase in neuronal excitability induced by abnormal cardiovascular conditions. However, it should be noted that E2 exerts different effects, depending on differing cardiovascular conditions. For instance, increased E2 levels during pregnancy induce hypertrophy, but E2 also attenuates the development of pressure-overload hypertrophy [22,50]. As the present study investigated E2-mediated modulation under normal conditions, different results can be expected under pathological conditions. This expectation is supported by the fact that different molecular targets were employed in the modulation of I_A_ in the PVN-RVLM neurons, depending on the modulating sources involved. A reduction in I_A_ in the PVN-RVLM neurons resulted from E2 replacement or artificial hypertension in a similar manner. Nevertheless, E2 down-regulated Kv4.2 mRNA levels, whereas hypertension down-regulated Kv4.3 [19]. Therefore, Kv4.2, as the target of E2 actions, could be modulated in different ways in PVN-RVLM neurons, according to normal or pathological conditions. Estrogenic effects on PVN-RVLM neurons modified by cardiovascular disorders remain to be determined.

## Conclusions

This study demonstrated that E2 modified the cellular properties of PVN-RVLM neurons. E2 played an inhibitory role in the I_A_ function in PVN-RVLM neurons and selectively down-regulated the mRNA expression of Kv4.2 but not that of Kv4.3. The finding that this modulation occurred predominantly in the DC subdivision suggests that functionally-discrete PVN-RVLM neurons may be distributed in specific subdivisions, indicating that these subdivisions deserve the important consideration in researching PVN-RVLM neurons. This study is the first evidence of estrogenic modulation of PVN-RVLM neurons.

## Competing interests

The authors declare that they have no competing interest or financial disclosures.

## Authors’ contributions

SKL performed the experimental design, work, data analysis and wrote the manuscript. PDR participate in its design and helped to draft the manuscript. SYL conceived the idea, designed experiments and edited the manuscript. All authors read and approved the final manuscript.
